# Antidiabetic and hypolipidemic activities of hydroethanolic root extract of *Uvaria chamae* in streptozotocin induced diabetic albino rats

**DOI:** 10.1186/s12906-016-1450-0

**Published:** 2016-11-15

**Authors:** Jonathan Emeka Emordi, Esther Oluwatoyin Agbaje, Ibrahim Adekunle Oreagba, Osede Ignis Iribhogbe

**Affiliations:** 1 Department of Pharmacology and Therapeutics, College of Medicine, Ambrose Alli University, Ekpoma, Nigeria; 2 Department of Pharmacology, Therapeutics and Toxicology, College of Medicine, University of Lagos, Lagos, Nigeria

**Keywords:** Diabetes mellitus, Hypoglycemic effects, Hypolipidemic effects, Streptozotocin, *Uvaria chamae*

## Abstract

**Background:**

Diabetes mellitus is a metabolic disorder of multiple aetiology characterised by hyperglycemia resulting from defects in insulin secretion, insulin action or both. It is a global epidemic ravaging both developed and developing countries. The situation will worsen if nothing is done urgently. In fact, the need to identify natural products with antidiabetic potentials is of great importance as supported by several research efforts all over the world, in search of antidiabetic plant based products that are safe and efficacious. Available literatures show that several phytochemicals with antidiabetic properties have been identified in certain plants amongst which include *Uvaria chamae*. The potentials of *Uvaria chamae* as an antidiabetic and hypolipidemic drug-candidate are thus tested.

**Methods:**

Diabetes mellitus was experimentally induced after the rats were fasted overnight by administering intraperitoneally, 60 mg/kg streptozotocin. After 72 h, the rats with plasma glucose levels >200 mg/dl were classified as diabetic. A total of six groups containing five rats per group were used. One group of diabetic rats was untreated. Three diabetic groups, each were treated orally with 100, 250 and 400 mg/kg body weight of the extract. Another diabetic group was treated with insulin (0.5 IU/kg) subcutaneously. The control received 0.5 ml (2% solution) of acacia orally. The treatment was for 8 days. The effects of the extract on weight, plasma glucose and other biochemical parameters were evaluated using standard procedures.

**Results:**

The diabetic rats treated with the extract showed significant reductions (*p* < 0.05) in weight, plasma glucose levels, low density lipoprotein and cholesterol compared with the control. The 100, 250 and 400 mg/kg body weight of the extract showed maximum glucose reduction of 85.16, 81.50 and 86.02% respectively. Histologically the pancreas of the diabetic rats treated with the extract, showed clusters of variably sized regenerated islet of Langerhans within sheets of normal exocrine pancreas, while the pancreas of diabetic rats treated with insulin showed no islet of Langerhans.

**Conclusion:**

The study showed that *Uvaria chamae* caused weight loss and has good hypoglycemic and hypolipidemic activities that may reduce the risk of developing cardiovascular diseases.

## Background

Diabetes mellitus has been described as a metabolic disorder of multiple aetiology, and characterized by chronic hyperglycemia with disturbances of carbohydrate, fat, and protein metabolism, resulting from defects in insulin secretion, insulin action or both [1, 2]. It is one of the oldest diseases of mankind that affects millions of people worldwide [3]. The number of people living with the disease is expected to double with major impact on the population of the developing countries due to increased rate of industrialization [3, 4]. It is a major risk factor for the development of cardiovascular disease [5]. Hence, the need to reduce the risk of vascular complications by securing adequate glycemic, lipidemic, blood pressure and weight control [6]. Currently diabetes is controlled by diet, exercise, oral hypoglycemic agents and insulin therapy [7]. The high level of treatment failures, unpleasant side effects and enormous cost associated with diabetic therapy have generated an urgent need and desire for alternative treatments [8]. However, the preferred choice of plant medicine by many might not be unconnected with the historical successes recorded in the use of herbal products in traditional system of medicine in the management of diabetes mellitus [9]. One of such plants used traditionally in the management of diabetes mellitus is *Uvaria chamae*.

*Uvaria chamae* is a medicinal plant that belongs to the family, *Annonaceae*. It is a climbing plant commonly found in West Africa [9]. In this region of the world, it is identified by numerous names such as: *Ogholo* by the Esan people of Edo state, *Ayiloko* by the Igalas, *Kaskaifi* by the Hausas, *Oko oja* by the Yorubas, *Mmimi ohia* by the Igbos in Nigeria and *Akotompo* by the Akan-fante people of Ghana [9]. The health benefits of medicinal plants are attributed in part to their unique phytochemical composition [10]. The phytochemical analysis of the leaves and roots of *Uvaria chamae* revealed the presence of alkaloids, glycosides, saponin, tannins, flavonoids, terpenoids and phenols [11, 12]. Our previous study on the preliminary phytochemical screening and evaluation of hypoglycemic properties of *Uvaria chamae* also revealed that the extract has secondary metabolites such as alkaloids, flavonoids, tannins and terpenoids which corroborate earlier works done on the phytochemical screening of this plant. The study showed that the hypoglycemic properties of *Uvaria chamae* may be accounted for by the presence of the phytochemicals [9]. The anti-fungal, anti-malarial and anti-inflammatory activities of *Uvaria chamae* have also been reported [11, 13, 14]. However, no scientific study has been conducted on the antidiabetic activity of this plant. The present study therefore was designed to evaluate the antidiabetic and hypolipidemic activities of the hydroethanolic root extract of *Uvaria chamae* in streptozotocin induced diabetic rats.

## Methods

### Plant materials

The roots of *Uvaria chamae* were obtained from a farm in Uromi, Edo State, Nigeria during the rainy season. They were authenticated by a taxonomist, Mr T. K. Odewo, of Department of Botany, University of Lagos, Nigeria. The voucher specimen with number LUH 3572 was deposited in the University herbarium.

### Preparation of the plant material for extraction

The roots were washed with clean water to remove foreign materials, chopped into small pieces and dried in an oven at 45° centigrade for 4 days. They were ground to coarse powder with electric grinder. The root powder, 500 g, was extracted with 93.3% hydroethanol by maceration with frequent stirring for 5 days. The extract was filtered using Whatman filter paper number 4 and concentrated with a rotary evaporator at a reduced pressure. The concentrated extract was dried in an oven at 40° centigrade to obtain 22.41 g dry residue (4.48% yields).

### Animals

Albino rats (160 ± 20 g) of both sexes were obtained from the Laboratory Animal Center, College of Medicine, University of Lagos, Idi-Araba and kept under standard environmental condition of 12/12 h light/dark cycle. They were housed in cages (5 animals per cage), maintained on standard animal pellets (Pfizer Feeds Plc, Nigeria), and provided with water *ad libitum.* They were allowed to acclimatize for 7 days to the laboratory conditions before the experiment. The use and care of the animals, and the experimental protocol were in strict compliance with the National Research Council guidelines on the care and use of laboratory animals [15]. The experimental protocol (with Protocol ID: RGEEC/21/2015) was approved by the research grants and experimentation Ethics committee of the College of Medicine, University of Lagos, Nigeria.

### Diabetic study

The dose selection for the diabetic study was guided by the result obtained from the oral acute toxicity study done in our previous work which showed that the median lethal dose was 7.08 g/kg body weight [9].

Diabetes mellitus was experimentally induced in the rats after an overnight fast by administering intraperitoneally (IP) 60 mg/kg streptozotocin dissolved in 0.1 M citrate buffer of PH 4.5 [16]. After 72 h, the blood glucose levels were monitored with a glucometer (*Accu-Chek*, Roche Diagnostics) and the rats with plasma glucose levels > 200 mg/dl were classified as diabetic [17] and were included in the study. A total of six groups containing five rats per group were used. Five groups were diabetic while the remaining group was used as a control. The rats were treated daily for 8 days orally except for those that were given insulin subcutaneously. The treatment was as follows:

- Group I: control given 0.5 ml (2% solution) of acacia
- Group II: Induced diabetic rats treated daily subcutaneously with (soluble) insulin 0.5 I.U / kg body weight
- Group III: Induced diabetic rats treated daily with 400 mg/kg of the extract
- Group IV: Induced diabetic rats treated daily with 250 mg/kg of the extract
- Group V: Induced diabetic rats treated daily with 100 mg/kg of the extract
- Group VI: Induced diabetic rats untreated

The rats were weighed and blood samples were collected from the tail vein for fasting blood glucose levels from the beginning of the treatment, the 5^th^ day and at the end of the treatment (the 8^th^ day). On the 8^th^ day, blood was obtained via ocular puncture into heparinised containers for biochemical profile.

### Sample analysis

The heparinised blood was centrifuged within 5 min of collection at 4000 g for 10 min to obtain plasma, that was analysed for total cholesterol (Chol), triglyceride (TG), and High density lipoprotein-cholesterol (HDL-cholesterol) levels by modified enzymatic procedures from Sigma Diagnostics [18]. Low density lipoprotein-cholesterol (LDL-cholesterol) levels were calculated using the Friedwald equation [19]. Plasma was analysed for alanine aminotransferase (ALT), aspartate aminotransferase (AST), alkaline phosphatase (ALP) and creatinine by standard enzymatic assay analysis [20]. The plasma protein contents and plasma glucose contents were determined using enzymatic spectroscopic methods [21].

### Tissue histology

The pancreatic tissue harvested from each group was fixed in 10% buffered formalin for 7 days before subjecting the tissues to routine histological processing techniques as described by Grizzle et al. [22] and staining with Haematoxylin and Eosin (H and E). Each section was examined under light microscope at high power magnification (×100 and x400) for structural changes. Photomicrographs were taken using an attached digital camera.

### Statistical analysis

Data analysis was done using Graph Pad Prism 6. One way analysis of variance (ANOVA) was used to compare means. One–way ANOVA was done followed by Dunnett’s multiple comparisons test of treated groups with control. The results were expressed as Mean ± SEM. Level of significance was set at *p* < 0.05.

## Results

### Antidiabetic activity of the root extract of *Uvaria chamae*

Table 1 is a summary of the results of the effect of the extract on the fasting blood glucose. There was an astronomical increase in the plasma blood glucose levels of the streptozotocin induced diabetic rats untreated compared with the control from day one to the last day of the experiment. There was also a significant (*p* < 0.05) increase in the plasma glucose levels of the diabetic rats treated with the extract and reference drug insulin compared with the control on day one. This showed that the rats were truly diabetic. However, on the 5^th^ and 8^th^ day (the last day of the experiment), there was a significant (*p* < 0.05) reduction of the plasma glucose levels of the diabetic rats treated with the extract compared with the control. While the plasma blood glucose levels of the diabetic rats treated with insulin was significantly (*p* < 0.05) increased compared with the control on the 5^th^ and 8^th^ day. The rats treated with 100, 250 and 400 mg/kg body weight of the extract showed a glucose reduction of 85.88, 80.00 and 85.48% respectively on the 5^th^ day. However, on the 8^th^ day of the treatment, rats treated with 100, 250 and 400 mg/kg body weight of the extract showed a maximum glucose reduction of 85.16, 81.50 and 86.02% respectively. While the rats treated with the reference drug insulin at the dose of 0.5 IU/kg body weight showed a glucose reduction of 25.43 and 63.28% on the 5^th^ and 8^th^ day respectively.

**Table 1:** Effect of *Uvaria chamae* on Fasting Blood Glucose (FBG) Levels (mg/dl) and % Reduction (% R) of FBG levels

Group	Day0	Day1	Day5	%R in Day5	Day 8	%R in Day8
I	74.33 ± 0.88	75.66 ± 2.19	86.00 ± 3.22	…….	90.67 ± 6.17	…….
II	59.33 ± 2.91	363.00 ± 50.5*	270.70 ± 33.17*	25.43	133.30 ± 10.04*	63.28
III	63.33 ± 0.67	303.00 ± 7.10*	44.00 ± 7.00*	85.48	42.37 ± 0.52*	86.02
IV	54.67 ± 1.45	263.30 ± 3.84*	52.67 ± 7.27*	80.00	48.70 ± 1.04*	81.50
V	66.67 ± 3.93	325.70 ± 17.85*	46.00 ± 2.08*	85.16	48.33 ± 0.88*	85.16
VI	60.00 ± 4.36	242.30 ± 3.93*	256.30 ± 1.86*	−5.66	300.30 ± 2.03*	−23.94

*Significant difference (*p* < 0.05; *n* = 5) between the Mean ± SEM of test group vs. control. Group I = (control) Normal rats received 0.5 ml (2% solution) of acacia, II = diabetic rats treated with insulin 0.5I.U/kg, III = diabetic rats treated with 400 mg/kg extract, IV = diabetic rats treated with 250 mg/kg extract, V = diabetic rats treated with 100 mg/kg extract, VI = diabetic rats untreated

### Effect of *Uvaria chamae* on plasma lipid profile

Table 2 is a summary of the results of the effects of *Uvaria chamae* on plasma lipid profile in diabetic rats. The plasma cholesterol and LDL levels of diabetic rats treated with the extracts were significantly reduced (*p* < 0.05) compared with the control. However, there was an increase in the HDL levels. The increase was not significant compared with the control. There was also a significant reduction (*p* < 0.05) in the plasma cholesterol and LDL levels of the diabetic rats treated with insulin. The plasma triglyceride levels of the diabetic rats untreated was significantly increased (*p* < 0.05) compared with the control.

**Table 2:** Effect of *Uvaria chamae* on Plasma Lipid Profile (mg/dl)

Parameter	Group I	GroupII	GroupIII	GroupIV	GroupV	Group VI
Chol	126.3 ± 2.3	78.0 ± 0.6*	72.3 ± 0.9*	70.0 ± 14.4*	89.33 ± 0.9*	107.0 ± 0.6
TG	43.2 ± 0.6	42.3 ± 1.9	57.3 ± 8.2	34.7 ± 0.3	61.3 ± 1.9	73.7 ± 0.9*
HDL	24.4 ± 0.4	25.3 ± 1.9	36.3 ± 4.1	31.7 ± 0.9	30.7 ± 0.7	29.7 ± 1.5
LDL	93.3 ± 0.6	44.2 ± 1.9 *	24.5 ± 7.8*	31.36 ± 7.13*	46.37 ± 1.53*	62.56 ± 2.52

*significant difference (*p* < 0.05; *n* = 5) between the Mean ± SEM of test group vs. control. Group I = (control) Normal rats received 0.5 ml (2% solution) of acacia, II = diabetic rats treated with insulin 0.5I.U/kg, III = diabetic rats treated with 400 mg/kg extracts, IV = diabetic rats treated with 250 mg/kg extract, V = diabetic rats treated with 100 mg/kg extracts, VI = diabetic rats untreated

### Effect of *Uvaria chamae* on other biochemical parameters

Table 3 is the summary of the result of the effects of *Uvaria chamae* on the other biochemical parameters. There was a significant decrease (*p* < 0.05) in the plasma protein levels of the diabetic rats treated with 400 mg/kg of the extract and the diabetic rats untreated compared with the control. The plasma albumin levels of the diabetic rats treated with insulin and 400 mg/kg of the extract were significantly reduced (*p* < 0.05) compared with the control. There was a significant increase (*p* < 0.05) in the plasma creatinine levels of the diabetic rats untreated compared with the control. The plasma urea levels of the diabetic rats treated with insulin, 400 mg/kg and 250 mg/kg of the extracts were significantly increased (*p* < 0.05) compared with the control. There was also a significant increase (*p* < 0.05) in the plasma urea level of the diabetic rats untreated compared with the control. There was no significant change in the plasma AST, ALT and ALP of the diabetic rats treated with the extract compared with the control. However, there was a significant increase (*p* < 0.05) in the plasma AST, ALT and ALP of the diabetic rats treated with insulin compared with the control. There was also a significant increase in (*p* < 0.05) the plasma AST and ALT levels of the diabetic rats untreated compared with the control.

**Table 3:** Effect of *Uvaria chamae* on other biochemical parameters on the 8^th^ Day

Parameter	Group I	Group II	GroupIII	GroupIV	GroupV	Group VI
Protein (g/L)	34.9 ± 0.1	25.7 ± 0.9	20.0 ± 2.3*	35.0 ± 1.2	23.7 ± 0.3	15.0 ± 2.0*
ALB (mg/dl)	43.9 ± 0.3	25.3 ± 1.5*	16.3 ± 1.5*	37.3 ± 0.9	27.0 ± 0.6	33.3 ± 1.2
Creatinine (mg/dl)	0.6 ± 0.01	1.0 ± 0.03	1.0 ± 0.1	1.03 ± 0.2	0.8 ± 0.1	1.13 ± 0.1*
Urea mg/dl	27.7 ± 0.9	56.0 ± 0.6*	64.0 ± 0.6*	43.3 ± 0.9*	31.0 ± 0.6	46.3 ± 2.3*
AST (U/L)	12.6 ± 0.2	27.2 ± 0.2*	13.3 ± 2.2	16.3 ± 0.3	13.8 ± 0.2	31.1 ± 1.8*
ALT (U/L)	10.8 ± 0.5	22.3 ± 1.3*	11.3 ± 2.2	14.8 ± 0.4	12.8 ± 0.4	27.7 ± 0.7*
ALP (U/L)	13.0 ± 0.3	30.7 ± 0.7*	14.7 ± 2.3	23.7 ± 5.7	16.7 ± 1.2	17.8 ± 0.03

*Significant difference (*p* < 0.05; *n* = 5) between the Mean ± SEM of test group vs. control. Group I = (control) Normal rats received 0.5 ml (2% solution) of acacia, II = diabetic rats treated with insulin 0.5I.U/kg, III = diabetic rats treated with 400 mg/kg extract, IV = diabetic rats treated with 250 mg/kg extract, V = diabetic rats treated with 100 mg/kg extract, VI = diabetic rats untreated

### Effect of *Uvaria chamae* on the body weight

The summary of the effects of the extract on the body weight of the rats is shown (Table 4). There was weight reduction in the rats treated with 100 and 250 mg/kg body weight of the extracts on the 5^th^ day. This weight reduction was not statistically significant. However, on the 8^th^ day there was a significant reduction (*p* < 0.05) in the weight of the rats treated with 100, 250 and 400 mg/kg body weight of the extracts compared with the control. The diabetic rats untreated showed a reduction in weight on the 5^th^ day. The weight reduction was significant (*p* < 0.05) on the 8^th^ day.

**Table 4:** Effect of *Uvaria chamae* on the body weight (g)

Group	Day1	Day5	Day8
I	130.7 ± 17.9	135.7 ± 20.5	159.0 ± 2.1
II	137.7 ± 5.8	127.3 ± 16.8	167.0 ± 3.5
III	133.7 ± 5.6	140.0 ± 6.1	122.7 ± 9.3*
IV	121.0 ± 4.4	107.7 ± 4.1	102.3 ± 1.5*
V	124.0 ± 7.2	118.3 ± 7.1	120.0 ± 2.9*
VI	135.0 ± 6.4	124.0 ± 2.7	118.3 ± 3.5*

*Significant difference (*p* < 0.05; *n* = 5) between the Mean ± SEM of test group vs. control. Group I = (control) Normal rats received 0.5 ml (2% solution) of acacia, II = diabetic rats treated with insulin 0.5I.U/kg, III = diabetic rats treated with 400 mg/kg extract, IV = diabetic rats treated with 250 mg/kg extract, V = diabetic rats treated with 100 mg/kg extracts, VI = diabetic rats untreated

### Histopathological studies

**Fig. 1:**
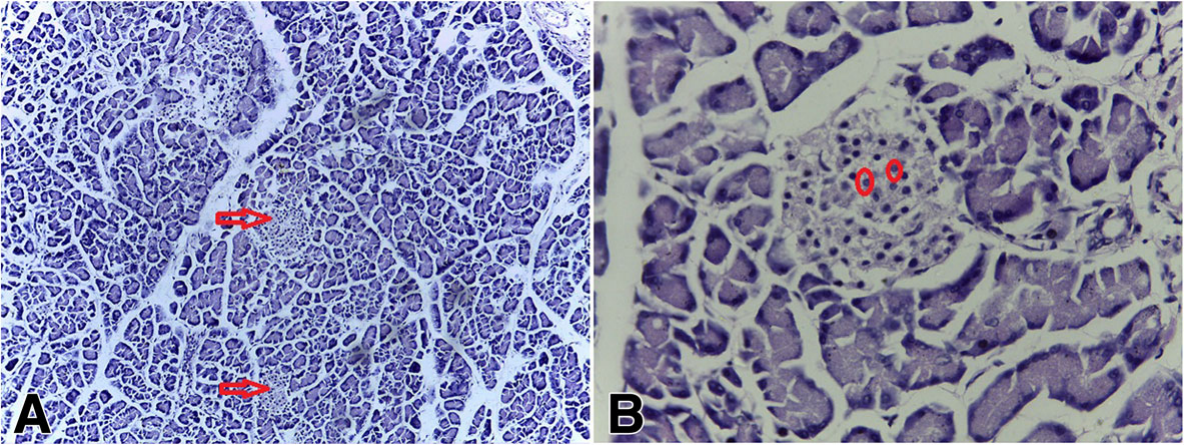
Photomicrograph of the pancreas of normal rat (positive control); H&E x100-A and x400-B showing Islet of Langerhans in plate **a** (*Red arrow*) and intact islet cells in plate **b** (*encircled*)

**Fig. 2:**
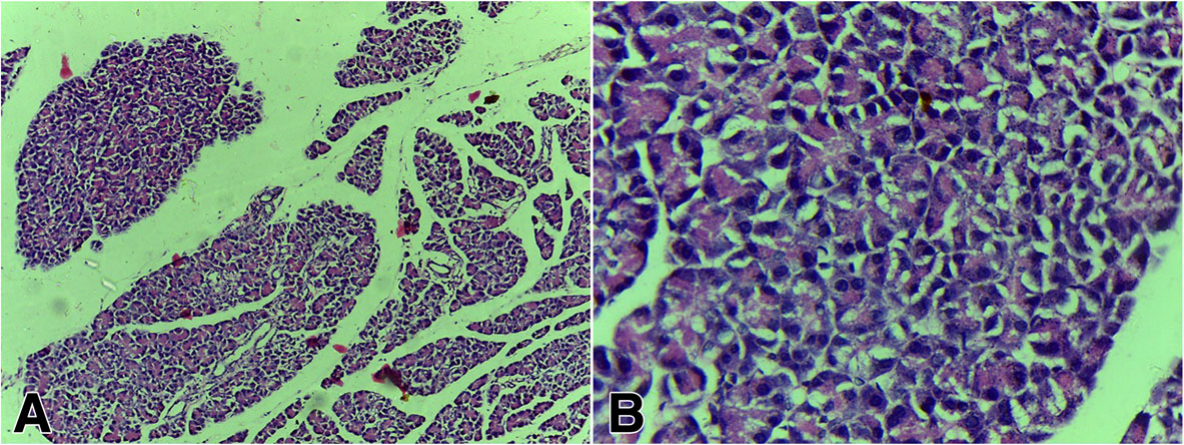
Photomicrograph of the pancreas of diabetic rat untreated (negative control); H&E x100 **a** and x400 **b** with no Islet of Langerhans

**Fig. 3:**
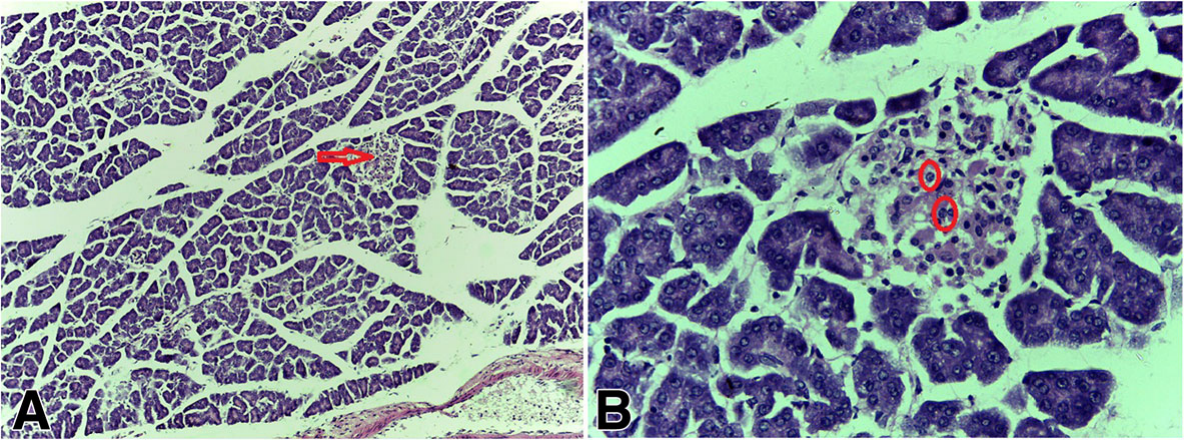
Photomicrograph of the pancreas of diabetic rat treated with 100 mg/kg of *Uvaria chamae* extract (H&E x100-A and x400-B) showing regenerated cells in the Islet of Langerhans (*See* plate **a**; *Red arrow*) and regenerated islet cells in plate **b** (*encircled*)

**Fig. 4:**
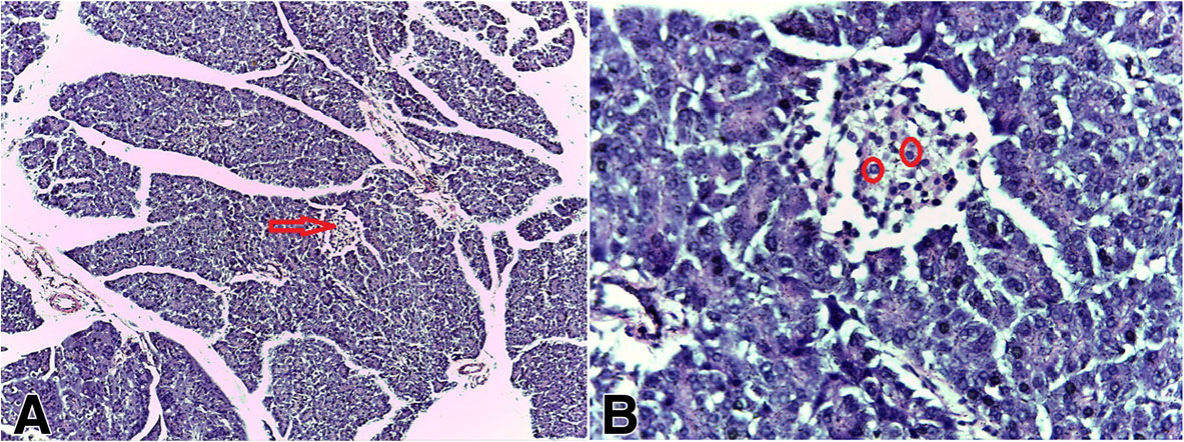
Photomicrograph of the pancreas of diabetic rat treated with 250 mg/kg of *Uvaria chamae* extract (H&E x100-A and x400-B) showing regenerated cells in the Islet of Langerhans (*See* plate **a**; *Red arrow*) and regenerated islet cells in plate **b** (*encircled*)

**Fig. 5:**
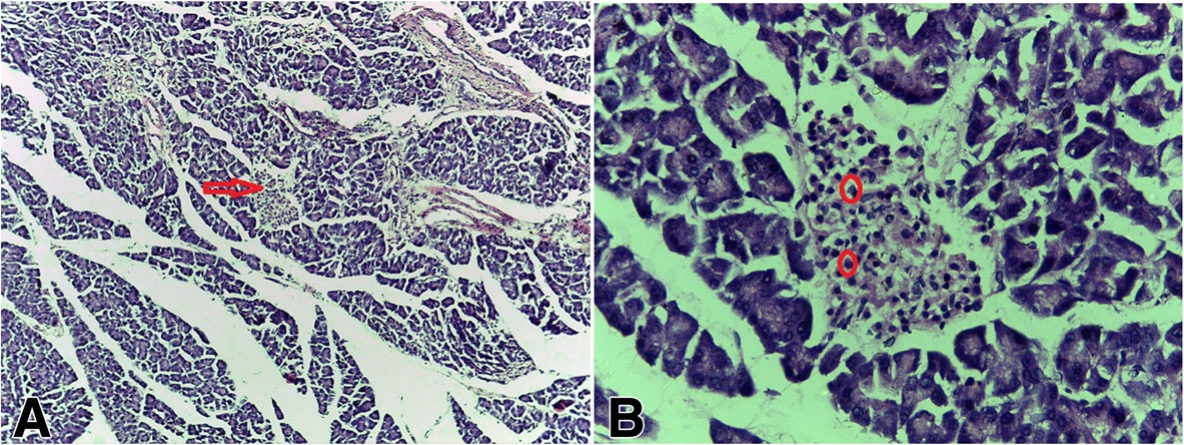
Photomicrograph of the pancreas of diabetic rat treated with 400 mg/kg of *Uvaria chamae* extract (H&Ex100-A and x400-B) showing regenerated cells in the Islet of Langerhans (*See* plate **a**; *Red arrow*) and regenerated islet cells in plate **b** (*encircled*)

**Fig. 6:**
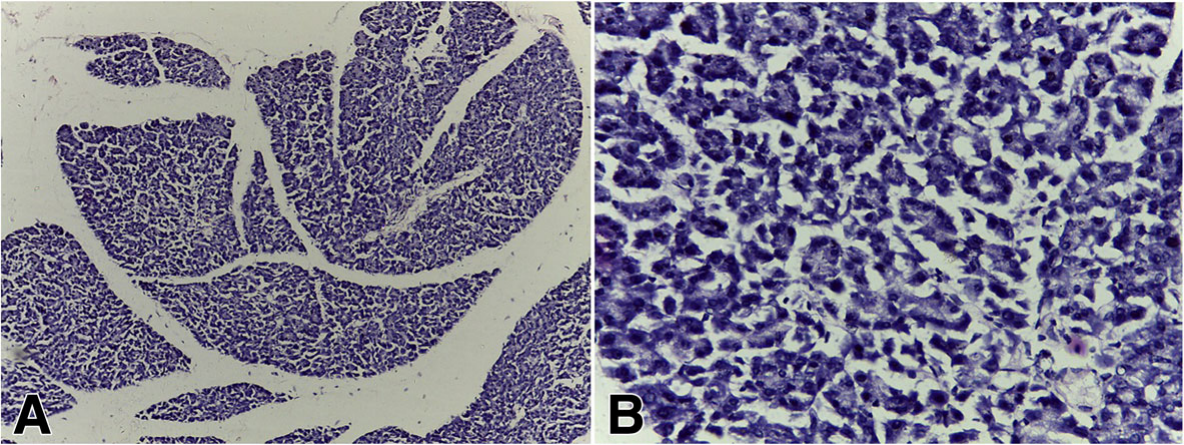
Photomicrograph of the pancreas of diabetic rat treated with 0.5 IU/kg of insulin (H&E x100-**a** and x400-**b**) showing no visible islets

The photomicrographs showing the histological features of the pancreas are shown in Figs. 1, 2, 3, 4, 5 and 6. The pancreas of the normal rats (Fig. 1) showed sheets of normal sized glands lined by columnar epithelium with basally located nuclei surrounded by vesicular cytoplasm. Interspersed within these glands are islands of normal sized Islet of Langerhans. The pancreas of the diabetic rats untreated (Fig. 2) showed sheets of small sized glands lined by columnar epithelium with basally located nucleus and vesicular cytoplasm. No Islet of Langerhan was seen. The pancreas of the diabetic rats (Figs. 3, 4, and 5), treated with the extract 100, 250 and 400 mg/kg body weight respectively, showed clusters of variably sized regenerated Islet of Langerhans within sheets of normal exocrine pancreas. However, the pancreas of the diabetic rats treated with insulin (Fig. 6), showed sheets of exocrine glands with parenchymal distortion. No Islet of Langerhan seen.

## Discussion

Diabetes mellitus is a disease that affects the quality of life and life expectancy of its victim’s worldwide [23]. Many deaths of diabetic subjects have been attributed to hyperglycemia and its accompanied vascular diseases. Hyperglycemia in particular, is the primary clinical manifestation of diabetes [24] and is thought to contribute to diabetic complications by altering vascular cellular metabolism, vascular matrix and circulating lipoproteins [25]. The major goal in the treatment of diabetes has been to keep both short-term and long-term glucose levels within acceptable limits, thereby reducing the risk of long term complications [26].

The result of this study showed that there was a significant reduction in the blood glucose levels of all the diabetic rats treated with the extract compared with the control. The 100, 250 and 400 mg/kg body weight of the *Uvaria chamae* extract achieved diabetic control at the 5^th^ day of treatment with a glucose reduction of 85.88, 80.00 and 85.48% respectively. This diabetic control was maintained till the end of the experiment. This marked reduction in plasma glucose concentration may be as a result of increased release of insulin from regenerated beta cells of the pancreas (Figs. 3, 4 and 5). The presence of phytochemicals such as flavonoids, alkaloids and tannins in the extract as reported in our previous study [9] may also have contributed to its antidiabetic activity.

Besides hyperglycemia, diabetes mellitus is highly characterized by elevated levels of triglycerides and cholesterol in the blood associated with a modern lifestyle and increased consumption of a high fat diet [27]. The reduced absorption of free fatty acids and free cholesterol by inhibition of pancreatic lipase and pancreatic cholesterol esterase reduces hyperlipidemia associated with diabetes mellitus [28, 29]. This study revealed that the extract lowered the plasma total cholesterol and LDL-cholesterol levels significantly in the treated diabetic rats. This clearly demonstrated the presence of hypolipidemic agents in the extract. The hypolipidemic activity of the extract may be due to the inhibition of pancreatic lipase and pancreatic cholesterol esterase. The reduced plasma LDL-cholesterol reduces the risk of developing cardiovascular disease [30].

Persistent hyperglycemia causes increase in cellular glucose level in tissues undergoing insulin-independent glucose uptake such as eye lens, retina, kidney, and peripheral nerves, leading to secondary late stage diabetic complications. Influx of excess glucose into polyol pathway causes accumulation of sorbitol in the tissues, resulting in hyperosmotic stress to the cells. This is postulated to be the primary cause of diabetic complications which include nephropathy, retinopathy, cataract, and neuropathy [31]. Creatinine is the most commonly used indicator of renal function. A raised plasma level of creatinine is a recognised marker of renal dysfunction [32]. The significant increase in the plasma creatinine levels of the diabetic rats’ untreated indicated renal impairment in this group of rats. Therefore, persistent hyperglycemia due to poorly controlled diabetes mellitus may lead to diabetic nephropathy. A number of extra renal factors influence the circulating urea concentration limiting its value as a test of kidney function. For example plasma urea concentration is increased by high protein diet, increased protein catabolism and dehydration. In the above pre-renal situations, the plasma creatinine concentration is usually normal [33]. The raised plasma urea seen in the diabetic rats treated with insulin and the extract could be as a result of dehydration or induced diabetic state from streptozotocin. This assertion is corroborated by Parvizi et al. [34], who reported that streptozotocin induced diabetes caused a significant increase in serum blood urea nitrogen level in type1 diabetes mellitus. ALT, AST and ALP are part of the liver enzymes [35]. They are frequently used to diagnose or screen for hepatobiliary disease, examine the progression of a disease as well as to monitor or detect the hepatotoxicity that may arise from the use of drugs or substances [36, 37]. The heart also releases AST and ALT, and an elevation in their plasma concentrations is an indicator of liver and heart damage [19]. The results in this study showed that the activities of AST and ALT in the plasma of diabetic rats untreated were markedly elevated. These enzymes are usually found in large quantities in the liver where they play an important role in the metabolism of amino acid [38]. However, as a result of damage or toxicity to the liver, these enzymes may leak from the hepatocytes into the circulation where their levels become elevated [39]. Therefore, the elevated plasma levels of AST and ALT in the diabetic untreated rats suggested liver and heart damage. Administration of the root extract of *Uvaria chamae* considerably reduced the elevated levels of AST and ALT in the diabetic rats. This showed that the extract is hepatocellular and cardio-protective. However, the elevated levels of AST, ALT and ALP in the diabetic rats treated with insulin during the period of the experiment could be as a result of damage to the liver and the heart from poorly controlled diabetes mellitus. Elevations of transaminases and alkaline phosphatase are common in diabetes mellitus [40]. Weight loss is considered an important aspect of therapy for patients with diabetes. Excess weight places greater direct demand on the beta-cell and also aggravates insulin resistance. Numerous studies have shown that weight loss in patients with diabetes can result in improvement in glucose levels [41]. The results of this study revealed that there was a significant weight reduction in the diabetic rats treated with the extract compared with the control. The weight loss may be due to suppression of appetite.

## Conclusion

This study showed that *Uvaria chamae* has antidiabetic and hypolipidemic activities. From the study the extract not only improved glycemic and lipidemic control but also caused weight loss, making it beneficial for diabetic patients that are overweight. These findings reduce the risk of developing cardiovascular disease and give credence to the use of the extract traditionally in the treatment of diabetes mellitus. The study also revealed that the extract is hepatocellular and cardio-protective.
